# Synaptic or Non-synaptic? Different Intercellular Interactions with Retinal Ganglion Cells in Optic Nerve Regeneration

**DOI:** 10.1007/s12035-022-02781-y

**Published:** 2022-03-09

**Authors:** Qi Zhang, Yiqing Li, Yehong Zhuo

**Affiliations:** State Key Laboratory of Ophthalmology, Zhongshan Ophthalmic Center, Guangdong Provincial Key Laboratory of Ophthalmology and Visual Science, Sun Yat-Sen University, Guangzhou, 510060 China

**Keywords:** Retinal ganglion cells, Optic nerve, Axon regeneration, Myelin, Glial scar, Neuroinflammation, Interneurons, Synapse

## Abstract

Axons of adult neurons in the mammalian central nervous system generally fail to regenerate by themselves, and few if any therapeutic options exist to reverse this situation. Due to a weak intrinsic potential for axon growth and the presence of strong extrinsic inhibitors, retinal ganglion cells (RGCs) cannot regenerate their axons spontaneously after optic nerve injury and eventually undergo apoptosis, resulting in permanent visual dysfunction. Regarding the extracellular environment, research to date has generally focused on glial cells and inflammatory cells, while few studies have discussed the potentially significant role of interneurons that make direct connections with RGCs as part of the complex retinal circuitry. In this study, we provide a novel angle to summarize these extracellular influences following optic nerve injury as “intercellular interactions” with RGCs and classify these interactions as synaptic and non-synaptic. By discussing current knowledge of non-synaptic (glial cells and inflammatory cells) and synaptic (mostly amacrine cells and bipolar cells) interactions, we hope to accentuate the previously neglected but significant effects of pre-synaptic interneurons and bring unique insights into future pursuit of optic nerve regeneration and visual function recovery.

## Introduction

Retinal ganglion cells (RGCs) play a central role in normal vision; their axons collectively form the optic nerve and extend through the chiasm, to innervate the lateral geniculate nucleus, superior colliculi, suprachiasmatic nucleus, and several other nuclei of the di- and mesencephalon [1]. The distant bridge from the retina to the brain renders the optic nerve vulnerable to injury, including traumatic and ischemic optic neuropathy, optic neuritis, and glaucoma, resulting in visual dysfunction and blindness [2]. Unfortunately, as with other central nervous system (CNS) pathways, RGCs have minimal intrinsic capacity to regenerate their axons after traumatic or ischemic injury or degeneration [3]. In addition, unlike the peripheral nervous system, multiple cell-extrinsic inhibitors of axon growth capacity also contribute to regenerative failure. Learning how to surmount these obstacles is the focus of most research aimed at achieving optic nerve regeneration [4, 5].

**Fig. 1:**
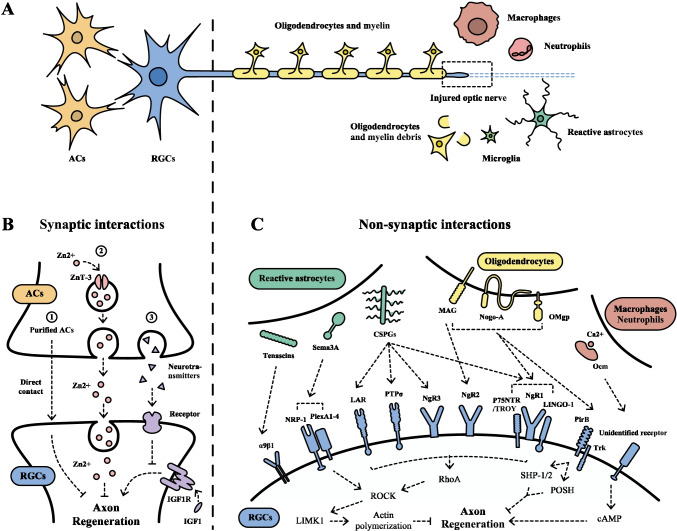
Schematic illustration of synaptic and non-synaptic interactions with retinal ganglion cells in optic nerve regeneration. (A) After optic nerve injury, various types of cells interact with RGCs and participate in optic nerve regeneration. These post-injury intercellular interactions could be classified into two broad categories: synaptic interactions, involving with ACs and BCs (not shown in the figure), and non-synaptic interactions, including glial cells (oligodendrocytes, reactive astrocytes, and microglia) and inflammatory cells (macrophages and neutrophils). (B) Synaptic interactions: (1) Purified ACs inhibit axon outgrowth through co-culture with RGCs in direct contact. (2) Mobile Zn^2+^ accumulated in ACs is transported in pre-synaptic vesicles by ZnT-3 and then transfer into RGCs through vesicular release to inhibit axon regeneration. (3) Inhibitory neurotransmitters released by ACs bind to post-synaptic receptors and attenuate axon regeneration induced by IGF-1. (C) Non-synaptic interactions: Nogo-A, MAG, and OMgp expressed by oligodendrocytes; CSPGs, Sema3A, and tenascins derived from reactive astrocytes; and Ocm secreted by macrophages and neutrophils all accumulate around damaged axons and bind to specific receptors on RGCs to inhibit axon regeneration. RhoA/ROCK/LIMK1 pathway is the primary signaling downstream of glial cell interactions and leads to subsequent actin polymerization and axon regeneration inhibition. PirB and Trk convey inhibitory signals of axon regeneration through two downstream cascades SHP-1/2 and POSH. The potent pro-regenerative effects of Ocm are mediated by increased level of intracellular cAMP. ACs, amacrine cells; BCs, bipolar cells; cAMP, cyclic adenosine monophosphate; CSPGs, chondroitin sulfate proteoglycans; IGF-1, insulin-like growth factor-1; LAR, leukocyte common antigen-related; LIMK, Lin-11, Isl-1 and Mec-3 kinase; LINGO-1, leucine-rich repeat immunoglobulin-like domain-containing protein 1; MAG, myelin-associated glycoprotein; NgR, Nogo receptors; NRP-1, neuropilin 1; Ocm, oncomodulin; OMgp, oligodendrocyte-myelin glycoprotein; PirB, paired immunoglobulin-like receptor B; PlexA1, plexin A1; POSH, Plenty of SH3s; PTPσ, protein tyrosine phosphatase sigma; p75NTR, p75 neurotrophin receptor; RGCs, retinal ganglion cells; ROCK, Rho-associated protein kinase; Sema3A, semaphorin 3A; SHP, Src homology 2-containing protein tyrosine phosphatase; Trk, tropomyosin receptor kinase; TROY, tumor necrosis factor receptor orphan Y; ZnT, Zn^2+^ transporter

Traditionally, when referring to the extrinsic environment of regenerating RGC axons, the spotlight is placed on myelin, the glial scar, and inflammation [6, 7], and not on the participation of other retinal neurons. However, the vital role of interneurons in RGC axon regeneration is receiving increasing attention lately. In this review, we summarize these extrinsic influences as “intercellular interactions,” a novel angle that discusses interactions after optic nerve injury between RGCs and other cells, including interneurons, glial cells, and inflammatory cells. For further description, we divide these interactions into two broad categories, namely synaptic and non-synaptic (Fig. 1).

In terms of the non-synaptic interactions, we mainly discuss the relationship between RGCs, glial cells, and inflammatory cells. On the one hand, myelin and glial scars, composed of inhibitory molecules from oligodendrocytes, reactive astrocytes, and microglia, have been demonstrated to mediate the majority of their interactions with RGCs and inhibit axon regeneration following optic nerve injury [7]. On the other hand, neutrophils and macrophages infiltrate the retina after injury and interact with RGCs via various cytokines and neurotrophic factors [8].

The synaptic interactions between RGCs and interneurons and their role in axon regeneration, however, remain a mystery. Previously, RGC death and regeneration were commonly considered to be cell-autonomous or influenced by glia. Recently, however, the importance of synaptic interactions between RGC and interneurons, mostly amacrine cells (ACs), has been realized [9]. Therefore, we summarize recent advances in the interneuron-mediated inhibition of axon regrowth and proposed several hypotheses regarding its potential mechanisms.

In general, we summarize current strategies to promote axon regeneration regarding non-synaptic interactions among retinal cells after optic nerve injury. Furthermore, we aim to draw attention to synaptic interactions between interneurons and RGCs, eventually pointing to a promising future for optic nerve regeneration.

## Non-synaptic Interactions with RGCs

### Oligodendrocytes and Myelin

Physiologically, myelin guarantees axon insulation, rapid conduction of electrical signals over distance, and metabolic support in the adult nervous system [10]. The myelin of the peripheral nervous system originates from Schwann cells, which provide a permissive environment for axon regeneration. While in the CNS, oligodendrocytes create myelin barriers for axon regeneration by expressing abundant inhibitory myelin-associated molecules [2, 3]. After optic nerve damage, transected or crushed axons are exposed to suppressed myelin-associated molecules. Prototypical myelin-associated inhibitors (MAIs) are mainly derived from oligodendrocytes, including Nogo, myelin-associated glycoprotein (MAG), and oligodendrocyte-myelin glycoprotein (OMgp) [11]. MAIs bind to their specific receptors on RGC axons and destabilize the actin cytoskeleton through intracellular downstream signaling, thereby collapsing axon growth cones and impeding axon regeneration [7, 12]. This process serves as the major intercellular interaction between oligodendrocytes and RGCs after optic nerve injury. Herein, we summarize the typical MAIs, their receptors, and related strategies to overcome this inhibition.

#### Typical Myelin-Associated Inhibitors

Nogo originates from the reticulon family and is expressed predominantly by oligodendrocytes in CNS [13, 14]. Among the three classical Nogo homologs (Nogo-A, Nogo-B, and Nogo-C) [15], Nogo-A has been identified as the dominant inhibitory component in oligodendrocytes and CNS myelin membranes, restricting axon regeneration ability of adult mammalian neurons [16–18]. In contrast, Nogo-B and Nogo-C found widespread expression in other tissue apart from the CNS [19]. Nogo-A is a multipass transmembrane protein with distinct molecular structures and growth-retrained regions. Several studies have identified different active sites of Nogo-A, such as NiG-Δ20 and Nogo-66, which are involved in growth cone destruction and neurite outgrowth inhibition [20–22].

MAG is the first identified neurite outgrowth inhibitor that belongs to the immunoglobulin gene superfamily [23] and is also selectively produced by oligodendrocytes in the CNS peri-axon membranes of myelin [24, 25]. Two forms of MAG polypeptides (72 and 67 kDa) expressed by a single transcript exist in myelin [26, 27]. MAG was found to bifunctionally regulate axon growth. Initially, researchers discovered that MAG facilitates interactions between glial cells and young neurons, ultimately enhancing neurite outgrowth [28]. However, subsequent studies have argued that MAG impedes neurite extension and activates growth cone retraction of older individuals [29]. Similarly, the influence of MAG on axon regeneration of adult CNS is controversial; the mainstream agrees with the inhibition of MAG, while others do not support its inhibitory role in axon regeneration [30].

OMgp is a glycosylphosphatidylinositol (GPI)-linked protein that is expressed by oligodendrocytes and neurons in the CNS [31–33]. In vitro studies revealed a highly conserved region of OMgp, the leucine-rich repeat (LRR) domain, which is necessary for growth cone collapse, neurite outgrowth inhibition, and cell proliferation [34]. Identical to Nogo and MAG, OMgp also contributes robustly to inhibitory activities associated with CNS myelin through distinct receptors and their attached complexes [35, 36].

#### Receptors of Typical Myelin-Associated Inhibitors

Nogo receptors (NgR) consist of the founding member NgR1 and its isoforms NgR2 and NgR3, which belong to the GPI-linked LRR protein family [37, 38]. Despite the highly heterogeneous structures of MAIs, they all bind to NgR with similar affinity [33, 39, 40]. The cross-activity of NgR partially stems from the overlapping binding domains of MAIs [41]. NgR1 is expressed by nearly all RGCs [42, 43] and exhibits a major function based on its intact intracellular signaling complex, involving the LRR immunoglobulin-like domain-containing protein 1 (LINGO-1) [44] combined with either p75 neurotrophin receptor (p75NTR) [45–47] or tumor necrosis factor receptor orphan Y (TROY) [48]. Later, NgR2 was found to bind MAG with a stronger affinity than NgR1 [49], while NgR3 may function as an NgR1 co-receptor [50]. Furthermore, paired immunoglobulin-like receptor B (PirB), associated with its partner tropomyosin receptor kinase (Trk), serves as another high-affinity receptor for MAIs in collaboration with NgR [51, 52]. Recent studies have implied the existence of PirB in the intact optic nerve and ganglion cell layer and its post-injury upregulation [53].

#### Interfering with Oligodendrocytes-RGCs Interactions to Promote Optic Nerve Regeneration

Starting with genetic interventions toward MAIs (Table 1), neurite sprouting and axon regrowth have been observed in triple knockout (Nogo-A, MAG, and OMgp) mice after injury [54, 55], while limited axon regeneration occurs when a single or double knockout (MAG and OMgp) is performed [30, 56], suggesting the potentially dominant role of Nogo-A and synergistic actions of MAG and OMgp. However, since Nogo-A is also expressed in neurons, including RGCs [19], subsequent experiments have shown that axon regeneration is not improved in traditional Nogo-A knockout mice, in which both glial and neuronal Nogo-A are deleted [57]. Recent studies indicate that Nogo-A expression in RGCs might enhance axon sprouting after injury and that specifically deleting Nogo-A in oligodendrocytes while preserving RGC Nogo-A could be a promising strategy to promote optic nerve regeneration [58]. Antibody-induced immunoblocking of MAIs also induces post-injury axon regeneration. Nogo-A antibodies, such as monoclonal antibody IN-1 [15], significantly increase axon regeneration in spinal cord injury [59, 60] and optic nerve crush (ONC) mice [61]. Similarly, the inhibitory effects on neurite sprouting are neutralized by MAG antibodies from the soluble fraction of myelin-conditioned media [62, 63].

**Table 1:** Interfering with oligodendrocytes-RGCs interactions to promote optic nerve regeneration

Targets		Mechanisms	Methods	In vitro/in vivo	Models	Outcomes	References
Ligands	Nogo-A/B/C	Nogo-A/B/C knockout	Transgenic mice	In vitro/in vivo	Cultured RGCs (mice)/optic nerve crush (mice)	Promote axon outgrowth /promote axon regeneration	[54]
	Nogo-A	Oligodendrocytes-specific Nogo-A knockout	Transgenic mice	In vivo	Optic nerve crush (mice)	Promote axon regeneration	[58]
	Nogo-A	Nogo-neutralizing antibody IN-1	Intravitreal injection	In vivo	Optic nerve crush (rats)	Promote axon regeneration	[61]
Receptors	NgR	RGC-specific NgR(DN) expression	AAV-NgR(DN)	In vivo	Optic nerve crush (rats)	Promote axon regeneration	[64]
	NgR1, 2, 3	NgR1, 2, 3 triple knockout	Transgenic mice	In vivo	Optic nerve crush (mice)	Promote axon regeneration	[65]
	NgR	RGC-specific NgR knockdown	Ocm/tp-NgR-siRNA	In vitro	Cultured RGCs (rats)	Promote axon outgrowth	[66]
	NgR, PirB	Endogenous NgR and PirB antagonist LOTUS overexpression	AAV-LOTUS	In vivo	Optic nerve crush (mice)	Promote axon regeneration	[67]
	p75NTR	Disturbing interaction between NgR1 and p75NTR by soluble LOTUS	Intravitreal injection	In vivo	Optic nerve crush (mice)	Promote axon regeneration	[68]
	PirB	PirB knockout	Transgenic mice	In vitro	Cultured neurons (mice)	Promote axon outgrowth	[51]
	PirB	RGC-specific PirB knockdown	AAV-PirB-siRNA	In vitro/in vivo	Cultured RGCs (rats)/optic nerve crush (rats)	Promote axon outgrowth /Promote axon regeneration	[69]
	NgR1	NgR1 competitive antagonist NEP1-40	Intravitreal injection	In vitro	Cultured RGCs (rats)	Promote axon outgrowth	[70]
	NgR1	NgR1 blocking protein NgR1(310)-Fc	Intravitreal injection	In vivo	Optic nerve crush (rats)	Promote axon regeneration	[71]
	PirB	PirB antibody	Co-cultured with neurons	In vitro	Cultured neurons (mice)	Promote axon outgrowth	[51]

*RGCs*, retinal ganglion cells; *NgR*, Nogo receptor; *NgR(DN)*, dominant negative form of NgR; *Ocm/tp*, oncomodulin/truncated protamine; *PirB*, paired immunoglobulin-like receptor B; *AAV*, adeno-associated virus; *p75NTR*, p75 neurotrophin receptor; *LOTUS*, lateral olfactory tract usher substance; *NEP1-40*, Nogo-A extracellular peptide 1–40

Manipulations toward receptors have also drawn attention. Although initial genetic deletion of NgR fails to inhibit neurite outgrowth in cultured neurons or promote axon regeneration in mice [72], subsequent experiments have demonstrated that NgR knockout alone is capable of inducing optic nerve regeneration at a moderate level [65, 73]. Transfecting RGCs with adeno-associated viruses (AAV) that express a dominant-negative form of NgR significantly stimulates axon regeneration after optic nerve damage [64]. Meanwhile, a competitive antagonist of NgR1, Nogo-A extracellular peptide (NEP1-40), can promote axon outgrowth in primary RGCs [70]. Intravitreal administration of an NgR blocking decoy, human NgR1(310)-Fc, successfully seals the receptors and regenerates axons in ONC mice [71]. Interfering with PirB activity, either genetically or with antibodies, also leads to partial relief from myelin inhibition, and simultaneously blocking NgR almost completely restores neurite outgrowth potentials in neurons cultured with myelin [51, 69]. Recently, the lateral olfactory tract usher substance (LOTUS), a newly discovered endogenous NgR [74] and PirB [75] antagonist, may become a potential therapeutic target. Studies have demonstrated that AAV-mediated overexpression of membrane-located LOTUS in RGCs blocks the binding between Nogo and NgR [67], and intravitreal injection of the soluble form of LOTUS suppresses the intracellular signal transduction of NgR1 by disturbing the connections between NgR1 and p75NTR [68], both of which significantly promote optic nerve regeneration in vivo.

#### Other Myelin-Associated Molecules

Semaphorin 4D (Sema4D), also known as CD100, is specifically expressed by oligodendrocytes and is transiently upregulated after optic nerve injury, serving as a novel inhibitory factor for axon regeneration [76]. Plexin B1, a receptor for Sema4D, induces repulsive responses by inactivating PI3K and dephosphorylating Akt and GSK-3β, triggering the collapse of growth cones and impeding axon regeneration [77]. Semaphorin 5A (Sema5A) is explicitly expressed by oligodendrocytes instead of astrocytes [78], and blockage of Sema5A by a neutralizing antibody significantly increases axon regrowth after injury [79, 80]. Ephrin-B3, previously identified as a repellant in axon guidance, accounts for inhibitory activity equivalent to that of the three main MAIs, further contributing to axon growth deficiency and regeneration limitation after CNS trauma [81, 82]. Netrin-1 is another axon guidance factor that is expressed by oligodendrocytes and binds its receptor complex DCC/UNC5 (namely deleted in colorectal cancer and uncoordinated-5) with a dual role during development [83] and inhibiting axon regeneration in the adult CNS [84, 85].

### Reactive Astrocytes and Glial Scarring

In response to injuries, the adult CNS initiates a rapid and protective response, also known as reactive astrogliosis or glial scarring, to repair and isolate tissue from secondary damage [86–88]. However, multiple studies have now shown that inhibitory molecule deposition in the scar contributes to the chemical barrier of axon regeneration, which is also the primary barrier [89–92]. Astrocytes, the glial cells that support synapse development, transmission, and plasticity [93], are thought to form molecular barriers of glial scar that prevent the post-injury regeneration of RGC axons [94, 95]. These inhibitory molecules have been demonstrated to include tenascins, semaphorins, ephrins, and, most importantly, chondroitin sulfate proteoglycans (CSPGs). Like MAIs, the inhibitory molecules deposited in the glial scar contribute to the interactions between reactive astrocytes and RGCs after injury by binding to their specific receptors.

#### Typical Inhibitory Molecules in the Glial Scar

CSPGs belong to a type of proteoglycans that consist of a protein core with adherent glycosaminoglycan (GAG) side chains [96] and serve as the predominant inhibitory components in the glial scar [97–99]. After optic nerve injury, CSPGs are secreted into the extracellular environment mainly by reactive astrocytes, oligodendrocytes, and macrophages [100, 101]. This secretion leads to a dense and persistent enrichment of CSPGs within the glial scar and specific inhibition of axon regeneration [102]. Administration of the chondroitinase ABC (ChABC) was shown to digest the GAG side chains and attenuate the inhibition of CSPGs [103], suggesting that the inhibitory properties of CSPGs could be attributed to the sulfated sugar GAG chains.

The semaphorin family contains eight classes, including secreted, membrane-associated, and GPI-anchored molecules, all of which conserve a specific “Sema” domain [104]. Accumulation of semaphorins within the glial scar is detected after mature mammalian central nerve injury [105]. Semaphorin 3A (Sema3A), a secreted molecule and a prototype of the semaphorin family, was initially discovered to guide axon growth in the development of the CNS [106]. Sema3A was later found to be upregulated after CNS injury [107], and several researchers have demonstrated that Sema3A is one of the dominant inhibitors of axon regeneration deposited in the glial scar [108, 109].

Tenascins belong to a family of oligomeric glycoproteins deposited in the extracellular environment [110]. The two archetypal glycoproteins of the tenascin family in vertebrates, tenascin-C (TN-C) and tenascin-R (TN-R), are upregulated during development and suppressed in the mature CNS [111], playing crucial roles in the development and pathology situation of the optic nerve. TN-C clumps in the glial scar secreted by reactive astrocytes and exhibits an inhibitory effect on axon regeneration after optic nerve transection [112] and other CNS injuries [113, 114]. Similar outcomes have been observed in goldfish after ONC [115]. TN-R has also been proposed to inhibit optic nerve regrowth and to persist at the lesion site in vitro [116, 117]. Interestingly, however, a shift in the expression levels of TN-R, either reduced or increased, was detected in salamanders [118] and lizards [119] after optic nerve injury.

#### Receptors of Inhibitory Molecules in the Glial Scar

Studies have indicated that the negative role of CSPGs in axon regeneration is predominantly mediated by two members of the leukocyte common antigen-related (LAR) phosphatase subfamily, transmembrane protein tyrosine phosphatase sigma (PTPσ) receptor and LAR phosphatase [120, 121]. In addition, NgR1 and NgR3 also serve as the functional receptors and mediate the anti-regenerative effects of CSPGs to some extent [65]. Receptors neuropilin 1 (NRP-1) and plexin A1 (PlexA1) serve as the functional co-receptors of Sema3A in neurons [122]. While NRP-1 acts as a binding segment, PlexA1 then activates its GTPase-activating protein domain and initiates downstream signaling pathways [123]. Integrin α9β1 is a transmembrane receptor with the ability to promote neurite outgrowth and axon regeneration [124, 125] when bound to the fibronectin type III domain of TN-C [126]. However, the expression of integrins is decreased in the adult nervous system [127] and even absent after CNS damage, particularly TN-C binding integrin α9β1 [128, 129], eventually eliminating the regenerative properties of TN-C and impeding axons from penetrating the glial scar.

#### Interfering with Astrocytes-RGCs Interactions to Promote Optic Nerve Regeneration

As stated above, treatments with ChABC attenuate CSPG inhibition and achieve RGC axon regeneration combined with other interventions in vivo [73] (Table 2). Initially, genetic deletion of PTPσ was found to diminish neuronal sensitivity toward CSPGs and comprehensively enabled regenerative RGC axons to penetrate the glial scar at the lesion sites [120, 130]. Some studies have revealed that optic nerve regeneration in NgR1 and 3 co-deficient mice was further enhanced when PTPσ was deleted [65]. Moreover, a peptide mimic of PTPσ binding to its wedge domain is sufficient to block CSPG-mediated inhibition in vitro, allowing adult neurons to regrow axons after injury [131]. Systemic administration of enoxaparin, a traditional anticoagulant, has been proposed to inactivate PTPσ and boost axon regrowth in rats with optic nerve injury at clinically tolerated doses [132]. Additionally, attenuation of excessive astrogliosis by microRNA (miR)-21 inhibition promotes axon regeneration and functional recovery of the flash visual evoked potentials (F-VEPs) in rats after optic nerve crush [133].

**Table 2:** Interfering with astrocytes-RGCs interactions to promote optic nerve regeneration

Targets		Mechanisms	Methods	In vitro/in vivo	Models	Outcomes	References
Ligands	CSPGs (GAG)	Digestion of GAG side chains by ChABC	Intravitreal injection	In vivo	Optic nerve crush (mice)	Promote axon regeneration	[73]
	Sema3A	Inhibition of Sema3A expression by miR-30b	AAV-miR-30b mimic	In vitro	Cultured RGCs (rats)	Promote axon outgrowth	[134]
	Sema3A	Inhibition of Sema3A expression by Sema3A siRNA	Lipofectamine induced Sema3A siRNA transfection	In vitro	Cultured RGCs (rats)	Promote axon outgrowth	[135]
Receptors	PTPσ	PTPσ knockout	Transgenic mice	In vivo	Optic nerve crush (mice)	Promote axon regeneration	[130]
	PTPσ	Inactivation and clustering of PTPσ by enoxaparin	Systemic administration	In vivo	Optic nerve crush (rats)	Promote axon regeneration	[132]
	NgR1, 3	NgR1, 3 double knockout	Transgenic mice	In vivo	Optic nerve crush (mice)	Promote axon regeneration	[65]
	α9β1	α9β1 and kindlin-1 expression in RGCs	AAV-α9β1 and kindlin-1	In vivo	Optic nerve crush (mice)	Promote axon regeneration	[136]

*CSPGs*, chondroitin sulfate proteoglycans; *GAG*, glycosaminoglycan; *ChABC*, chondroitinase ABC; *Sema3A*, semaphorin 3A; *RGCs*, retinal ganglion cells; *PTPσ*, protein tyrosine phosphatase sigma receptor; *NgR*, Nogo receptor; *AAV*, adeno-associated virus

Although intravitreal injection of anti-Sema3A antibodies improves RGC survival after optic nerve transection [137], none of its effects has been reported in axon regeneration. Therefore, researchers have diverted their attention away from antibodies to anti-expression. MiR-30b was found to inhibit Sema3A expression by binding to the 3′ untranslated region of Sema3A mRNA; transfecting cultured RGCs with AAV-miR-30b reduced Sema3A expression levels and significantly promoted the growth of axons while impeding the growth of dendrites [134, 135]. Likewise, Sema3A small interfering RNA and miR-30b overexpression exert similar effects on axon regeneration, collectively representing a new target for the treatment of optic nerve injury [135]. Moreover, inhibition of Sema3A intracellular signaling transduction alleviates the suppressed axon regeneration and completely rescues the decreased amplitude of F-VEPs induced by Sema3A [138].

In addition to CSPGs and Sema3A, studies of TN-C- or TN-R-deficient mice with spinal cord injury showed increased amounts of neural fibers penetrating the glial scar [139]. However, others argue that TN-C is necessary for axon regeneration as more axons retract after injury in TN-C knockout mice, and this retraction could be rescued via viral-mediated overexpression of TN-C [140]. Further studies have observed improved axon regeneration and functional recovery via polyclonal antibodies against TN-R [116]. Additionally, re-expression of TN-C after injury, along with the integrin activator kindlin-1, promotes neurite outgrowth and axon regeneration in the spinal cord [141] and optic nerve [136].

### Controversy in Microglia

Microglia are resident immune cells originating from macrophages in the mammalian CNS and play an essential role in both physiological homeostasis maintenance and pathological immune responses [142, 143]. Physiologically, microglial cells constantly monitor the CNS environment and scavenge cellular debris, DNA fragments, and infectious agents [144, 145]. Upon sensing injury, microglia are immediately activated and release cytokines and neurotrophic factors, leading to macrophage infiltration and immune defensive responses [146, 147]. However, whether microglial activation is beneficial or detrimental during CNS repair after damage remains unclear [148]. Microglia can induce neurotoxicity and aggravate the subsequent neurodegeneration after injury, but they can also contribute to the protective mechanisms of tissue repair and regeneration, depending on the characteristics of insults as well as microenvironmental conditions [149]. On the one hand, activated microglia are reported to produce neurotrophic factors and eliminate destructive debris that eventually promotes neuron survival and axon regeneration [150–152]. On the other hand, unidentified inhibitory molecules and numerous inflammatory cytokines can impede axon regrowth and damage the intact neurons near the injury site [153–155].

According to the macrophage activation process [156], microglia can be generally divided into at least two subclasses with distinct functions depending on the activation pathway. M1 microglia are “classically activated” by lipopolysaccharides or interferon γ (IFN-γ) and then produce large amounts of oxidative metabolites and proinflammatory cytokines, resulting in a defensive immune response, astrogliosis reactivation, and neuronal damage. In contrast, M2 microglia are “alternatively activated” by interleukin-4 (IL-4) and IL-13, and they express IL-10 and arginase-1, collectively downregulating inflammation and promoting tissue repair [142]. An elevated M1/M2 ratio leads to secondary neurodegeneration in mice after spinal cord injury, while a shift in microglial phenotypes from M1 to M2 was observed during the wound healing and axon regeneration processes [157–159].

After optic nerve injury, microglia become activated and infiltrate at the lesion site, recruiting macrophages, phagocytosing cellular and axonal debris, and even becoming involved in glial scar formation [160–162]. However, the distinct interactions between microglia and RGCs after optic nerve injury remain unclear. We have little idea of the neurotoxic or neuroprotective effects of activated microglia on RGCs in response to damage. Our previous work showed that laquinimod, a newly discovered immunosuppressant, impeded microglia activation and attenuated high intraocular pressure-induced RGC death [163], and that long non-coding RNA-H19 served as a crucial progenitor of microglia pyroptosis and RGC death after ischemia/reperfusion-induced inflammation [164]. It is unknown whether optic nerve regeneration is enhanced or suppressed by cytokines and neurotrophic factors secreted by microglia. The controversial and insufficiently identified characteristics of microglia could be partially attributed to the lack of reliable experimental methods to distinguish microglia from other invading immune cells [165]. Recently, a study reported a novel inhibitor of colony stimulator factor 1 receptor (CSF1R) that could efficiently eliminate more than 99% of microglia without affecting macrophages and other immune cells [166]. Interestingly, after intravitreal injection of this inhibitor, the extent of RGC degeneration after optic nerve injury remained unaffected, and no significant alterations were detected in RGC axon regeneration induced by lens injury. Slight but significant inhibition of optic nerve regeneration was observed when microglia and macrophages were co-depleted [167]. Therefore, a new theory is proposed that, despite their role in macrophage recruitment and phagocytosis, microglia may not be necessary for RGC degeneration and axon regeneration after acute optic nerve injury.

### Inflammatory Cells and Neurotrophic Factors

After injury-induced inflammation, macrophages and neutrophils are the primary immune cells that are recruited and accumulate within the lesion site; then, they interact with RGCs via the secretion of various cytokines and neurotrophic factors that manipulate the axon regeneration process. Intraocular inflammation, induced by a lens injury or intravitreal zymosan injection, can delay injury-induced RGC degeneration and promote axon regeneration beyond the optic nerve lesions, profoundly influencing neurological outcomes [6, 168, 169].

#### Macrophages

Macrophages have been shown to change the non-permissive environment to a pro-regenerative environment at the lesion sites of adult rat optic nerve in vitro [170]. Later, an elevated level of infiltrated macrophages was detected after lens injury or zymosan injection, accompanied by protein accumulation later identified as oncomodulin (Ocm) [171]. Ocm is a calcium-binding protein mainly derived from activated macrophages and neutrophils [8, 172]. After intraocular inflammation, Ocm increases within the retina and binds to RGCs with high affinity in a cAMP-dependent manner, serving as a potent growth factor and stimulating significant axon regrowth in vitro and in vivo [173, 174]. Combinatorial treatment with Ocm and cAMP analogs simulates the pro-regenerative effects of intraocular inflammation, while peptides or antibodies against Ocm almost neutralize the positive effects of zymosan injection on optic nerve regeneration [174]. In addition, Ocm has been used as a guide in a vector complex delivering small interfering RNA of NgR to RGCs because of its high affinity, which dramatically promotes axon regrowth of RGCs [66].

#### Neutrophils

Neutrophils are reported to be immediately activated and accumulate within lesions after spinal cord injury [175]. Shortly after intraocular zymosan injection, the vast majority of neutrophils infiltrate the eyes before macrophages, and they promote optic nerve regeneration by expressing high levels of Ocm [8]. Immunodepletion of neutrophils reduces the expression of Ocm within the eyes and suppresses inflammation-induced regeneration. Anti-Ocm intervention abolished axon regeneration as effectively as neutrophil depletion. Moreover, macrophages are insufficient to induce axon regeneration in the absence of neutrophils, implying that neutrophils might play a vital and pro-regenerative role in inflammation-induced axon regeneration [8]. Meanwhile, the combined deletion of the pattern recognition receptors dectin-1 and toll-like receptor 2 (TLR2) completely abolished the effects of zymosan injection, and further intravitreal injection of the dectin-1 ligand curdlan promotes axon regeneration at the same level as zymosan [176]. Recently, a study has found that administration of Ly6G-specific antibodies successfully depleted neutrophils and significantly compromised the pro-regenerative effects of neurotrophic factors [177]. Another study identified a unique subset of immature neutrophils (CD14 + Ly6Glo) induced by inhibition of C-X-C motif chemokine receptor 2 (CXCR2), which promotes axon regeneration in part via the secretion of various growth factors [178].

#### Neurotrophic Factors

Ciliary neurotrophic factor (CNTF) is elevated after injury or zymosan-induced intraocular inflammation [179] and serves as a leading therapeutic candidate to promote neuroprotection and axon regeneration after CNS injury [180, 181]. However, recombinant CNTF (rCNTF) has been shown to have limited effects on axon regeneration [182]. The low efficacy of rCNTF could be attributed to the upregulation of the suppression of cytokine signaling factor 3 (SOCS3), an inhibitor of the JAK/STAT pathway, in mature RGCs [168, 182, 183]. After SOCS3 knockout, rCNTF is capable of enhancing regeneration by activating gp130-dependent kinase signaling [179]. However, another therapeutic method, AAV-mediated CNTF expression, promotes robust optic nerve regeneration by itself, and this pro-regeneration effect involves neuroinflammation, which is mediated primarily by C–C motif chemokine ligand 5 (CCL5) instead of direct action on RGCs [177]. Leukemia inhibitory factor (LIF) has also been identified as an additional contributing factor to CNTF, as CNTF and LIF combined knockout completely abolishes the positive effects on RGC survival and axon regeneration [179, 184]. Interestingly, the role of brain-derived neurotrophic factor (BDNF) post-injury is relatively controversial. After optic nerve injury, BDNF protects RGCs from cell death [185], but also attenuates the level of inflammation-induced axon regeneration [186].

## Synaptic Interactions with RGCs

As concluded above, the physiological functions and pathological impacts of glial cells and inflammatory cells after optic nerve injury were thoroughly evaluated. However, we ignored another large number of cells that also have inseparable connections with RGCs, namely interneurons. Due to their neuronal characteristics, the interactions between interneurons and RGCs are predominantly mediated via synapses, which are significantly different from non-neurons. Recently, several studies have concentrated on excavating the role of interneurons and their synaptic impacts on RGC axon regeneration.

Retinal interneurons, consisting of horizontal cells, bipolar cells (BCs), and ACs, play an essential role in the normal retina. In mammalian retinal circuitry, photoreceptors translate light energy into neural signals and convey them to interneurons. Within the outer plexiform layer, horizontal cells contact photoreceptors and mediate feedback and feedforward inhibition of photoreceptors and BCs, respectively [187]. In the inner plexiform layer, BCs form excitatory synapses with at least 40 distinct types of ganglion cells [43, 188], and various ACs regulate these connections through pre-synaptic and post-synaptic inhibition [189, 190]. Given the evidence that only BCs and ACs have direct contact with RGCs, and their vital role in normal retinal circuitry, subsequent studies have mainly focused on the effects of these two types of interneurons after optic nerve injury.

### Amacrine Cells

ACs are very diverse predominant inhibitory interneurons in the retina circuit that diminish RGCs’ activity state through inhibitory neurotransmitters, including glycine, γ-aminobutyric acid (GABA), and dopamine [191, 192]. Nearly all ACs form inhibitory synapses with RGCs and could be divided into two subclasses: glycinergic narrow-field type and GABAergic wide-field type [193]. True to their name, instead of axons, ACs output signals to BCs, RGCs, and other ACs via the same dendrites that receive synaptic inputs [187].

The exploration of interneuron-associated axon regeneration inhibition began with ACs. Initially, Goldberg and colleagues found that purified ACs could induce neonatal RGCs to irreversibly transform their growth mode from axonogenesis to dendritogenesis in culture. This inhibition of axon growth occurred only when RGCs and ACs were co-cultured in direct contact. Therefore, they proposed that a contact-mediated or membrane-associated signal plays a role in this inhibition [194, 195]. Although this conclusion merely stopped at the surface of this phenomenon, it provided a hint that this inhibition, unlike that from glial cells, could be involved with synapses since membranes are necessary. After a decade, direct evidence of synaptic interactions has been proposed. Ionic zinc (Zn^2+^) was reported to accumulate in the AC processes immediately after ONC and keep increasing during the first 24 h, and then transfer into RGCs through synaptic vesicular release, thereby impeding axon regeneration [196]. Recently, optic nerve injury pathologically activates ACs, which later reduces the electrical activity and growth factor responsiveness of RGCs. Genetically silencing ACs or blocking inhibitory neurotransmitters both enhance RGC growth factor signaling, thereby promoting optic nerve regeneration [197].

### Bipolar Cells

BCs receive various information, output excitatory signals to a diversity of neuron types in the retina, and, most importantly, connect with photoreceptors, primary sensory neurons, and RGCs, which project their axons out of the eye to the brain [198, 199]. The first operations and analysis of visual systems occur in BCs, which collect photoreceptor signals and accept adjustment from horizontal and ACs for further processing in the inner retina [200].

In a retinal regeneration model of zebrafish, after intravitreal injection of ouabain, a cytotoxin that destroys the inner retina, BCs, and their post-synaptic partner RGCs were shown to survive and regenerate concurrently [201]. Intriguingly, RGCs are generated prior to BCs in the embryonic retina of zebrafish, which is different from the sequence of neurogenesis in regeneration [202]. Furthermore, morphological measurements of BC axons found that the thickness of the inner plexiform layer and number of axon branches were slightly reduced after regeneration, but the synapses on their axons were in excess of the usual number, suggesting a certain impact of BCs on RGC axon regeneration [203, 204]. In an optic nerve injury situation, it has been proposed that BCs are involved in RGC protection and axon regeneration indirectly through interactions with ACs. Excitatory neurotransmitter glutamate released by BCs binds to NMDA and AMPA receptors on the post-synaptic membrane of ACs, leading to the latter neurons’ activation, depolarization, Ca^2+^ influx, and eventually Zn^2+^ accumulation [9]. In fact, AC-specific blockage of NMDA receptors suppresses mobile Zn^2+^ elevation within AC processes after ONC [9]. Nevertheless, the direct effects of BCs on axon regeneration still remain unclear.

### Potential Mechanisms of Synaptic Interactions in Optic Nerve Regeneration

After uncovering the inhibitory effects of interneurons after optic nerve injury, we investigated the potential mechanism behind this phenomenon. Herein, we summarize the results of previous related experiments and propose several hypotheses.

#### Antagonistic Axon-Dendrite Interplay

In adult zebrafish, a vertebrate animal model that is capable of spontaneously regenerating CNS axons, synapse degeneration, and dendrite retraction was immediately observed in RGCs after ONC. Axon regeneration was initiated only when remarkable synapse and dendrite shrinkage occurred [205]. Intriguingly, dendritic regrowth and reconnection occurred after the regenerative axons reinnervated their target neurons in the optic tectum [205].

This obvious time-organized sequence of dendrite remodeling and axon regrowth in zebrafish suggests an antagonistic axon-dendrite interplay after ONC. Hence, several studies have focused on whether the inhibition of dendrite shrinkage promotes axon regeneration. Inhibition of mTOR via intravitreal injection of rapamycin successfully preserved synapses and dendrites early after ONC, which subsequently restrained axon regeneration and reinnervation [206]. However, if the administration of rapamycin was delayed until the synapses and dendrites had already deteriorated, no signs of negative impact on axon regeneration were observed [206]. Together, these data could indicate an underlying antagonistic interplay between axon regrowth and dendrite remodeling and that dendrite shrinkage is favorable for axon regeneration during CNS repair.

In this context, it is reasonable that dendrite shrinkage after axon injury is the necessary preparation for axon regrowth in mammals, despite their failure of regeneration due to the lack of intrinsic potentials and strong inhibitory environments. Some evidence supports this opinion. As mentioned above, the contact-mediated or membrane-associated signals from ACs irreversibly switch neonatal RGCs from an axon growth mode to a dendrite growth mode [194]. Intravitreal injection of rCNTF combined with cAMP analogs or Rho-GTPase inhibitor (BA-210) has been shown to increase RGC survival and axon regeneration [207, 208]. However, this pro-regenerative outcome is accompanied by aberrant dendrite morphologies, including excessive looping processes and shrinking but sparser dendritic fields [209]. Similarly, dendrites have been proposed to repress axon regeneration through a dual leucine zipper kinase (DLK)-independent pathway, as cutting dendrites in the DLK knockout background relieve the anti-growth signal and result in axon regeneration [210]. Apart from RGCs, axon regeneration of light and pheromone-sensing neurons (ASJ) in *Caenorhabditis elegans* is enhanced when axotomy is performed simultaneously with dendritomy compared to axon transection alone [211].

Overall, the retraction of RGC dendrites from their synaptic connections with interneurons has been proven to benefit axon regeneration and nerve repair. Nevertheless, more research is clearly needed to provide solid evidence for the observed antagonistic axon-dendrite interplay and the role of synaptic inhibition during optic nerve regeneration.

#### Inhibitory Neurotransmitters

The diversity of neurotransmitters that carry information between neurons within their synaptic clefts provides the nervous system with remarkable complexity and functionality [212, 213]. In the retina circuitry, glycine, GABA-A, and dopamine receptors are distributed on RGC dendrites and receive inhibitory input from ACs [214, 215], whereas glycine and GABA-C receptors are distributed on BC axon terminals that also mediate inhibitory signals from ACs [216, 217]. Hence, it is plausible that these inhibitory neurotransmitters and their receptors are critical therapeutic targets after optic nerve injury.

A massive amount of aminoacidergic neurotransmitters (mostly glycine, GABA, and glutamate) has been observed after spinal cord injury [218, 219]. Excessive accumulation of glutamate and glycine results in excitotoxicity and neuronal loss [220–222]. In contrast, GABA has been reported to promote survival and regeneration of descending neurons after spinal cord injury in larval lampreys [223]. Unfortunately, few studies have demonstrated the pro-regenerative effects of these neurotransmitters in mammals, and none of these results has been verified in optic nerve injury models. Nevertheless, some studies have indirectly hinted at the influence of inhibitory neurotransmitters on optic nerve regeneration. After ONC, a drug cocktail consisting of antagonist blockers of inhibitory neurotransmitter receptors was immediately injected into the vitreous body of mice. The responsiveness of RGCs to growth factors was enhanced, and axon regeneration level was significantly increased when antagonist cocktail treatment was combined with insulin-like growth factor-1 (IGF-1) [197]. Intriguingly, the recovery of signaling competence toward IGF-1 was caused by the maintenance of IGF-1 receptors in RGC primary cilia, which would have been lost upon optic nerve injury [197].

In another view, the level of RGC physiological activity depends on the excitatory or inhibitory neurotransmitters from pre-synaptic interneurons, which also alters the growth state of RGCs. Thus, different experiments were conducted to explore the role of neuronal activity in axon regeneration. In cultured immature RGCs, BDNF-induced axon outgrowth was remarkably potentiated by spontaneously generated electrical activity at physiological levels [224]. After ONC, RGC axon regeneration is enhanced by elevated levels of electrical activity induced by light stimulation through GPCR signaling [225] or by transcorneal electrical stimulation [226, 227]. Recent findings indicate that increasing mouse RGC activity by visual stimulations or chemogenetics combined with mTOR activation could serve as a potent tool for improving axon regeneration, even with partial recovery of visual functions and vision-guided behaviors, including the optomotor response, pupillary response, depth perception, and circadian entrainment [228, 229]. Upon activation and depolarization of RGCs, Ca^2+^ influx then initiates active-dependent transcription and elevates the level of cAMP, which in turn promotes the expression of growth-related genes, bridging the gap between neuronal activity and pro-regenerative function [230, 231].

All these facts taken together, we still need to uncover the precise role of each kind of neurotransmitter, either protective or neurotoxic, in the optic nerve injury situation. Further, more in-depth studies are required to determine the specific receptors that neurotransmitters bind and how intracellular post-synaptic signaling downstream is mediated.

#### Other Synaptic Vesicular Contents

In addition to neurotransmitters, other vesicular contents transported within synaptic clefts may contribute significantly to the synaptic interactions between interneurons and RGCs post-injury. As stated above, Zn^2+^ was reported to increase in ACs immediately after ONC and then transfer into RGCs through synaptic vesicular release. Zn^2+^ transporter 3 (ZnT-3) knockout in *slc30a3*-deficient mice significantly attenuated Zn^2+^ accumulation in the AC vesicles and eventually promoted axon regeneration. Intravitreal administration of Zn^2+^ chelators also promoted axon regeneration and even enhanced the pro-regenerative effects of phosphatase and tensin homolog (PTEN) and Krüpple-like factor (KLF)-9 knockout [196, 232].

AC-specific nitric oxide (NO) production could be the vital progenitor of mobile Zn^2+^ accumulation within ACs after optic nerve injury. Upon RGC axon injury, ACs are initially hyperactivated followed by Ca^2+^ influx [197], which in turn initiates NO generation through activating the NO synthetase [233–235]. Reactive nitrogen species are then produced and induce intracellular oxidative stress, thereby leading to the liberation of Zn^2+^ from metallothioneins and elevation of mobile Zn^2+^ in ACs [236, 237].

### Current Puzzles and Future Directions of Synaptic Interactions

As stated above, the majority of the experimental outcomes supporting the hypotheses were from retinal regeneration models of non-mammals, which have not been proven in mammals with optic nerve injury. That is to say, more studies are needed to provide more solid theories of the mechanisms behind synaptic interaction after optic nerve injury. Moreover, it seems feasible and promising to draw lessons from other CNS pathways. Some research has shown that the transected axons from mammalian spinal interneurons could spontaneously regenerate [238]; therefore, elucidating the underlying mechanisms that allow these axons to regrow might enlighten us on some new hypotheses. Additionally, we still could not fully understand how ACs are instantly hyperactivated after the axons of RGCs are injured. A theory is proposed that Cl^−^ gradient dysregulation in the interneurons after optic nerve injury leads to positive feedback circuits between BCs and ACs, which in turn results in early hyperactivation of ACs [9, 239]. Apart from ACs, little is known about the role of BCs in axon regeneration. How do they respond to optic nerve injury? How do they interact with RGCs under traumatic circumstances? More answers are needed to the above-mentioned and subsequent questions.

## Conclusion

To date, we have already identified abundant extracellular molecules, such as Nogo, MAG, OMgp, CSPGs, semaphorins, tenascins, and many neurotrophic factors; they all bind to specific receptors on RGCs, the process that is classified as “non-synaptic interactions.” Various interventions targeting these ligands or their receptors have been shown to promote axon regeneration over past decades. Meanwhile, we intend to focus more on the role of “synaptic interactions” in optic nerve regeneration. Interfering with the synaptic connections between ACs and RGCs has been shown to be a powerful strategy to enhance axon regenerative ability. We have already proposed several theories that may elucidate this process to a certain extent. The exploration of different intercellular interactions with RGCs after optic nerve injury could help us better understand the initial and subsequent responses in the complex retinal circuit and lead to new therapeutic strategies for axon regeneration.

## Prospects

Remarkable progress in RGC axon regeneration has been achieved over the past two decades, and the optic nerve is now considered one of the principal models for axon regeneration investigation in the CNS. However, compared with the rapid progress achieved in basic research, few clinical treatments have been proved to markedly rescue certain forms of blindness in patients. A great deal of obstacles remains to be overcome to achieve clinically meaningful visual recovery.

One of the greatest challenges is the lack of long-distance axon regeneration since functional visual recovery is based on an adequate amount of RGC axons regenerating across the optic chiasm and reinnervating specific targets in the brain [240]. In this regard, more research should focus on axon guidance signals that are capable of navigating axon growth during development or after injury (e.g., ephrins, netrins, and semaphorins) [84, 241–243]. In addition, combined treatments have always been found to promote a more substantial regeneration effect than individual treatments [240]. The next challenge is to extend the therapeutic window in clinical practice by increasing RGC survival rate while promoting axon regeneration. Axon regeneration apparently requires intact RGC soma with full function, especially in the case of glaucoma, when RGC degeneration and irreversible loss are key factors in the pathological process. Preliminary studies have uncovered various methods to protect RGCs from neuron death in rodent models of acute and chronic glaucoma [244–246]. Furthermore, it is of great necessity to find optimized animal models that could better bridge the gap of clinical translation. Currently, the rodent ONC model is one of the most widely used animal models for RGC survival and axon regeneration research. However, this surgically easy and highly reproducible animal model cannot fully imitate complicated clinical conditions related to optic nerve injury. Nonhuman primate models could be the solution to this problem. A growing number of researchers have been dedicated to uncovering the molecular and cellular processes that underline nonhuman primates in various retina and optic nerve diseases through recently developed multi-omics approaches [247–249].

Collectively, the field of optic nerve regeneration and vision recovery is not only challenging but also full of hope. Transplantation of RGCs is starting to emerge as a realistic approach to reverse certain forms of blindness. Researchers attempt to replace damaged RGCs after optic nerve injury with neonatal ones differentiated from embryonic stem cells or induced pluripotent stem cells, and promising progress has been achieved [250–256]. Even the theory of RGC transplantation from deceased donors into recipients’ eyes has been supported in rats with successful integration and axon projection after allogeneic RGC transplantation [257]. Promisingly, both basic and translational studies possess great potential to achieve long-distance axon regeneration and full recovery of visual function for some time to come.

## Data Availability

Not applicable.
